# Preventing β-amyloid fibrillization and deposition: β-sheet breakers and pathological chaperone inhibitors

**DOI:** 10.1186/1471-2202-9-S2-S5

**Published:** 2008-12-03

**Authors:** Thomas Wisniewski, Martin Sadowski

**Affiliations:** 1 Department of Neurology, New York University School of Medicine, 550 First Avenue, New York, NY 10016, USA; 2 Department of Pathology, New York University School of Medicine, 550 First Avenue, New York, NY 10016, USA; 3 Department of Psychiatry, New York University School of Medicine, 550 First Avenue, New York, NY 10016, USA; 4 Department of Pharmacology, New York University School of Medicine, 550 First Avenue, New York, NY 10016, USA

## Abstract

Central to the pathogenesis of Alzheimer's disease (AD) is the conversion of normal, soluble β-amyloid (sAβ) to oligomeric, fibrillar Aβ. This process of conformational conversion can be influenced by interactions with other proteins that can stabilize the disease-associated state; these proteins have been termed 'pathological chaperones'. In a number of AD models, intervention that block soluble Aβ aggregation, including β-sheet breakers, and compounds that block interactions with pathological chaperones, have been shown to be highly effective. When combined with early pathology detection, these therapeutic strategies hold great promise as effective and relatively toxicity free methods of preventing AD related pathology.

## Introduction

Formation of β-amyloid (Aβ) fibrils and deposition of Aβ in the brain parenchyma, or in the brain's vessels, occurs in the setting of increased Aβ peptide concentrations [1,2]. Initially, conditions do not favor aggregation of fibrils, but once a critical nucleus has been formed, conditions change to favor aggregation in an exponential manner. Any available monomer becomes instantly entrapped in an aggregate or fibril. Several compounds – for example, Congo red [3], anthracycline [4], rifampicin [5], anionic sulphonates [6], and melatonin [7] – can interact with Aβ and prevent its aggregation into oligomers and fibrils *in vitro*, reducing toxicity. These oligomeric structures have been associated with the greatest toxicity [8]. Several Aβ homologous peptides have been identified that have amino acid substitutions using residues such as proline and can bind to Aβ oligomers and fibril structures, leading to disruption of the β-sheet conformation [9-12]. These peptides have been termed β-sheet breakers. An advantage of such compounds, in comparison to other putative therapeutic approaches for AD, such as vaccination, is that they specifically target the abnormal conformation of Aβ and will not disrupt any possible normal function of the soluble Aβ peptide. Several modifications have been used to extend the serum half-life and increase the blood-brain barrier (BBB) permeability of these β-sheet breakers. Permanne *et al. *[13], using a BBB permeable pentapeptide (iAβ5), were able to demonstrate a reduction of Aβ load in AD Tg mice compared to an age-matched control group. Of interest, a similar concept of β-sheet breakers appears to be applicable to other protein conformational disorders caused by prions [14].

## Pathological chaperone inhibitors

Aβ homologous peptides can spontaneously aggregate and form fibrils *in vitro*; however, *in vivo *this process appears more dependant on Aβ pathological chaperones. This group of proteins actively promotes conformational transformation by increasing the β-sheet content of these disease-specific proteins, stabilizing their abnormal structure [15-17]. Examples in Alzheimer's disease (AD) include apolipoprotein E (apoE), especially its E4 isoform [16,18], α1-antichymotrypsin [19], and C1q complement factor [20,21]. In their presence, the formation of Aβ fibrils in a solution of soluble Aβ monomers becomes much more efficient [16,19]. These 'pathological chaperone' proteins have been found histologically and biochemically in association with fibrillar Aβ deposits [15,22-24], but not in preamyloid aggregates, which are not associated with neuronal toxicity [25-27]. Inheritance of the apoE4 isoform has been identified as the major genetic risk factor for sporadic, late-onset AD [28] and correlates with an earlier age of onset and greater Aβ deposition in an allele-dose-dependent manner [28,29]. On the other hand, epidemiological data suggest that inheritance of the E2 allele has a protective effect. *In vitro *all apoE isoforms can propagate the β-sheet content of Aβ peptides promoting fibril formation [16,23], with apoE4 being the most efficient [16]. The critical dependence of Aβ deposition in plaques on the presence of apoE has also been confirmed in AD Tg amyloid precursor protein (APP)^V717F^/apoE^-/- ^mice, which have a delayed onset of Aβ deposition, a reduced Aβ load, and no fibrillar Aβ deposits compared with APP^V717F^/apoE^+/+ ^Tg mice. APP^V717F^/apoE^+/- ^mice demonstrate an intermediate level of pathology [30-33]. Neutralization of the chaperoning effect of apoE would therefore potentially have a mitigating effect on Aβ accumulation. ApoE binds hydrophobically to amino acids 12–28 of Aβ, forming SDS-insoluble complexes [34-36]. Ma *et al. *[37] have demonstrated that a synthetic peptide homologous to this sequence of Aβ can be used as a competitive inhibitor of the binding of full length Aβ to apoE, resulting in reduced fibril formation *in vitro *and increased survival of cultured neurons. Several modifications to Aβ12–28, including the replacement of a valine for proline at position 18 (Aβ12–28P), made this peptide non-toxic, non-fibrillogenic, and prevented any potential for co-deposition on existing Aβ plaques. Further modifications included protection of its amino and carboxyl termini, and using D-amino acids resulted in an extended serum half-life (62 ± 18 minutes, mean ± standard deviation. These modifications did not limit its ability to block the apoE-Aβ interaction (*K*_*i *_= 11.37 nM) [38,39]. Although Aβ12–28P had a limited serum half-life, it was able to cross the BBB, exerting a therapeutically prolonged effect. Treatment of APP^K670N/M671L^/PS1^M146L^, and APP^K670N/M671L ^AD Tg mice with Aβ12–28P resulted in a significant reduction of Aβ deposition in brain parenchyma and in brain vessels [39]. Furthermore, treatment with Aβ12–28P prevented memory decline in single APP Tg mice. Measurement of Aβ levels in the brain homogenate revealed a significant reduction in the absolute Aβ level while the concentrations of the soluble Aβ fraction and Aβ oligomers remained stable during treatment [39]. This observation is important in light of a concern regarding the blocking of the apoE-Aβ interaction and that this form of therapeutic interference could destabilize fibrillar Aβ assemblies, and impair apoE-mediated Aβ clearance, potentially increasing brain levels of soluble Aβ, thus favoring the formation of toxic oligomers [8,40]. During treatment with Aβ12–28P, no signs of toxicity, including systemic amyloidosis or disturbance of vascular integrity leading to cerebral hemorrhages (as observed with some vaccination approaches [39]), were noted. The treatment outcome with Aβ12–28P could not be attributed to an immunization effect since there were no significant changes in the serum titer of anti-Aβ antibodies before and after the treatment of AD Tg animals. Thus, interference with the apoE-Aβ interaction *in vivo *appears to have a net effect of increasing Aβ clearance and reducing the absolute Aβ level in the brain, producing a cognitive benefit. Further development of ligands inhibiting the apoE-Aβ interaction, potentially making them suitable for clinical application, should be aggressively pursued.

Of interest, Pepys and co-workers [41] have developed a ligand that chelates serum amyloid-P component. Serum amyloid-P component, along with apoE, binds to amyloid fibrils *in vivo *in amyloid-A primary systemic amyloidosis, and inhibits the normal degradative process of amyloid-A [42]. Both disease model animals and affected humans treated with this compound in phase I/II clinical trials demonstrated reduced amyloid-A deposition [41].

A similar treatment concept is being developed based on inhibiting the interaction between glycosaminoglycans and Aβ fibrils [6]. Neurochem Inc. is conducting clinical trials to determine the efficacy of 3-amino-1-propanesulfonic acid (3APS), a synthetic sulphated glycosaminoglycan mimetic designed to compete with naturally occurring glycosaminoglycans in their binding to Aβ to prevent its deposition . The efficacy of this compound is being tested for AD and cerebral amyloid angiopathy under two different brand names, Alzhemed™ and Cerebril™, respectively. The phase II clinical trial of Alzhemed™ (tramiprosate) demonstrated a good safety profile, with the most frequently reported adverse events being nausea and vomiting, the occurrence of which appeared to be dose related. This multi-centre, double-blind, placebo-controlled study recruited 58 patients who received either tramiprosate (100 mg, 200 mg or 300 mg daily) or placebo. Results of this trial indicated that tramiprosate reduced the Aβ1–42 concentration in the cerebrospinal fluid and stabilized cognitive decline (monitored using the Mini-Mental State Examination (MMSE) and Alzheimer's Disease Assessment Scale-cognitive subscale (ADAS-cog) scales) compared with the control group [43]. Unfortunately, the North American phase III clinical trial of tramiprosate, which finished in late 2007, did not provide clear enough benefit in a large population. The clinical data gathered by this trial are being subjected to secondary statistical analysis. Currently, the prospect of FDA approval for tramiprosate remains unclear.

Another class of compounds characterized by inherent anti-Aβ aggregation properties *in vivo *comprises the cyclohexanehexol (inositol) stereoisomers. These compounds are naturally occurring sugars characterized by good gastrointestinal absorption, making them attractive candidates for clinical development. When administered orally to AD Tg mice, the cyclohexanehexol stereoisomers scyllo-cyclohexenehexol and epi- cyclohexenehexol reduced several disease features, including impaired cognition, altered synaptic physiology, and cerebral Aβ deposition [44]. Clinical trials of scyllo-cyclohexanehexol are currently entering a multi-center phase II trial under the name ELND005.

The US phase II clinical trial of NC-758 (Cerebril™) for the prevention of hemorrhagic stroke due to cerebral amyloid angiopathy has recently finished. This multi-center, randomized, double-blind and parallel-designed study was conducted at five centers, enrolled 24 cerebral amyloid angiopathy (CAA) patients, and found no safety concerns related to the use of NC-758. Neurochem Inc. has also recently successfully finished a phase II/III clinical trial of 1,3-propanedisulfonate (Fibrillex™), a compound designed to treat amyloidosis A, which is associated with a number of systemic, chronic inflammatory diseases, including rheumatoid arthritis and Crohn's disease. Its mechanism of action is analogous to that of tramiprosate. Based on the results of this trial the company has filed for market approval.

## Necessity for early diagnosis and treatment

For maximal benefit, it is likely that all of the interventions discussed above will need to be initiated at the earliest stages of AD pathology [45]. From AD models, therapeutic strategies to prevent and remove amyloid deposition using methods such as vaccination are very effective at preventing cognitive deficits when initiated at the time deposition first starts. However, when the same treatment is administered after deposits are well-established there are no cognitive benefits [46]. In AD patients it is estimated that mild cognitive impairment slowly develops over 10–30 years prior to the presentation of pathology [47,48]. In the active vaccination study, which targeted early clinical stage AD, patients showed a very modest cognitive benefit [49,50] despite the fact that autopsies on four study participants indicated that the vaccine had produced a significant reduction in amyloid plaques [51]. Positron emission tomography (PET) imaging of amyloid binding ligands can detect amyloid deposition at early stages [52] and similar methods are being developed for amyloid detection by magnetic resonance imaging (MRI) [53]. These methods will be critical in identifying patients who are at risk of developing clinical AD and who might benefit from preventing Aβ aggregation and deposition therapies.

## Conclusion

Numerous therapeutic interventions are currently being developed for AD. It is likely that, in the future, multi-modal therapies will be tailored to individual patients based on their genotype, immune status, and pathological stage of their disease. However, β-sheet breakers and pathological chaperone inhibitors are likely to be among the most effective and safest strategies.

## List of abbreviations used

Aβ: β-amyloid; AD: Alzheimer's disease; apoE: apolipoprotein E; APP: amyloid precursor protein; BBB: blood-brain barrier; CAA: cerebral amyloid angiopathy.

## Competing interests

The authors declare that they have no competing interests.

## Authors' contributions

Both authors carried out a literature review. TW wrote the draft, which was modified and corrected by MS.
